# Evaluation of the Biological Activity of Hydrogel with *Cornus mas* L. Extract and Its Potential Use in Dermatology and Cosmetology

**DOI:** 10.3390/molecules28217384

**Published:** 2023-11-01

**Authors:** Martyna Zagórska-Dziok, Aleksandra Ziemlewska, Agnieszka Mokrzyńska, Zofia Nizioł-Łukaszewska, Magdalena Wójciak, Ireneusz Sowa

**Affiliations:** 1 Department of Technology of Cosmetic and Pharmaceutical Products, Medical College, University of Information Technology and Management in Rzeszow, Sucharskiego 2, 35-225 Rzeszow, Poland; aziemlewska@wsiz.edu.pl (A.Z.); amokrzynska@wsiz.edu.pl (A.M.); zniziol@wsiz.edu.pl (Z.N.-Ł.); 2 Department of Analytical Chemistry, Medical University of Lublin, Aleje Raclawickie 1, 20-059 Lublin, Poland; magdalena.wojciak@umlub.pl

**Keywords:** *Cornus mas* L., skin cells, hydrogels, antioxidants, skin aging, collagenase, elastase, skin hydration

## Abstract

Due to the growing popularity of herbal extract-loaded hydrogels, this study assessed the biological activity of extracts and hydrogels containing three types (water (WE), water–ethanol (EE) and water–glycerin (GE)) of *Cornus mas* L. (dogwood) extracts. The content of biologically active compounds in the extracts was assessed using the UPLC-DAD-MS technique. Antioxidant properties were assessed by using DPPH and ABTS radicals and measuring the intracellular level of reactive oxygen species. Alamar Blue and Neutral Red tests were used to measure the cytotoxicity of the tested samples on skin cells—fibroblasts and keratinocytes. Cell migration and the anti-aging activity of the tested extracts and hydrogels were assessed. Transepidermal water loss and skin hydration after applying the hydrogels to the skin were also determined. A chromatographic analysis revealed that the extracts contained polyphenols, including gallic, caftaric, protocatechuic, chlorogenic, ellagic and p-coumaroylquinic acids, as well as iridoids, with loganic acid as the predominant component. Additionally, they contained cyanidin 3-*O*-galactoside, pelargonidin 3-*O*-glucoside and quinic acid. The obtained results show that the tested extracts and hydrogels had strong antioxidant properties and had a positive effect on the viability of skin cells in vitro. Additionally, it was shown that they stimulated the migration of these cells and had the ability to inhibit the activity of collagenase and elastase. Moreover, the tested hydrogels increased skin hydration and prevented transepidermal water loss. The obtained results indicate that the developed hydrogels may be effective delivery systems for phytochemicals contained in dogwood extracts.

## 1. Introduction

The skin, as the largest organ of the human body, is constantly exposed to many environmental factors, such as UV radiation, temperature fluctuations, various chemical factors and mechanical injuries [1]. Maintaining its healthy appearance is mainly based on its care, counteracting the negative effects of various factors and treating skin lesions and wounds resulting from various skin disorders. The most common skin diseases are primarily acne, bacterial and fungal infections, eczema, atopic dermatitis, psoriasis and rosacea [2]. Additionally, biologically active compounds that could eliminate unpleasant skin sensations, such as burning, itching and dryness, are being sought. Many medical preparations have been developed for this purpose, but skin diseases are a serious problem for people all over the world. Currently, new biologically active substances with a broad spectrum of action that could prevent skin imperfections as well as treat and eliminate skin lesions are being searched for [3]. Invaluable ingredients of many cosmetic and pharmaceutical preparations intended to eliminate skin lesions are plant extracts, which are extremely rich sources of many biologically active compounds. These multi-component mixtures of biomolecules have been used for centuries in folk medicine to eliminate many skin lesions. The extraordinary diversity of different groups of compounds in plant extracts enables their potential use in the treatment of skin diseases of various origins. A great advantage of using plant extracts in cosmetic recipes is that they have fewer side effects, including irritations and allergies, compared with synthetic ingredients. As a result, consumers are increasingly willing to buy cosmetic preparations based on natural raw materials, which may have multidirectional effects [3,4]. Therefore, the development of cosmetic preparations containing extracts full of bioactive phytochemicals may contribute to a significant improvement in the condition of the skin. These extracts can both perform a care function and be effective ingredients of cosmetics, both influencing the biological functions of the skin and providing a wide spectrum of various nutrients for healthy skin [5].

*Cornus mas* L. (dogwood) is a species of shrub or small tree from the *Cornaceae* family mainly growing in Eastern Europe, Southern Europe and the Middle East [6]. It is a plant with ovate or oblong leaves and drupes of various colors. The part of the plant that is commonly used is primarily dogwood fruit, which is eaten fresh, dried or pickled. Jams, tinctures, marmalades and juices obtained from the fruit of this plant are also very popular [7]. Numerous scientific articles describe the versatile properties of *C. mas* L., which include the treatment of fever, kidney stones, urinary tract infections, diarrhea and heat stroke [8]. Additionally, it has been shown that this plant can help regulate blood glucose levels, improve lipid profile parameters, have a positive effect on the cardiovascular system and be helpful in the treatment of atherosclerosis and some types of cancer [8,9]. Extracts obtained from various parts of this plant also have antioxidant and anti-inflammatory properties and may also be helpful in preventing bacterial and parasitic infections. The latter properties indicate that this plant may be a valuable tool in the fight against skin diseases accompanied by oxidative stress, inflammatory reactions and microbiological infections. This effect is related to the presence of numerous phytochemicals in dogwood extracts, such as catechin, epicatechin, gallic acid, ellagic acid, quinic acid, chlorogenic acid, loganic acid, quercetin, kaempferol and pelargonidin, the interaction of which is responsible for the final therapeutic effect [9,10]. 

In recent years, hydrogel materials have become very popular as carriers of active substances in cosmetology and dermatology. These matrices can significantly improve the effectiveness of the delivery of a wide spectrum of phytochemicals and thus positively influence the effects on the treatment of various diseases, including skin diseases [11]. Hydrogels are three-dimensional polymer networks in the structure of which many therapeutic compounds, both hydrophilic and hydrophobic, can be incorporated. Therefore, they can be used in the treatment of many diseases requiring the use of therapeutic compounds with different structures and physico-chemical properties [12]. Progress in the design of intelligent hydrogel matrices has made it possible to change their volume and controlled release of the compounds after stimulation with appropriate external stimuli, such as changes in pH, temperature, pressure, electrical signals and various biochemical molecules [13]. The increased interest in hydrogels as delivery systems for therapeutic substances is largely related to the simplicity of their application and the minimization of side effects due to local, topical drug delivery achieved by applying the hydrogel to the skin surface. This method of application allows one to obtain the desired therapeutic concentrations of biologically active compounds in individual layers of the skin without affecting their concentration in the serum [14]. Consumers primarily choose hydrogels based on natural polymers (carboxynethyl cellulose, alginate, chitosan, carrageenan, hyaluronan) due to their low toxicity, high biocompatibility, physical properties similar to natural tissues and the possibility of biodegradation [15]. These matrices are, therefore, used primarily for the treatment of various dermatological diseases accompanied by various types of local changes on the skin surface. Various types of hydrogels have been developed for the treatment of skin lesions occurring in many skin diseases, such as psoriasis, mycoses and acne vulgaris [7]. Additionally, hydrogels are increasingly used as multifunctional biomaterials in tissue engineering and cell therapy, as biosensors and as useful tools in regenerative medicine [11,16]. There are difficulties in using bioactive compounds of plant origin as therapeutic agents due to their limited bioavailability and often low solubility in water, but their incorporation into hydrogel structures can solve this problem and have a positive impact on the effects on the treatment of skin lesions. Additionally, the possibility of drug release controlled by these biopolymers, as well as the possibility of incorporating natural plant compounds and synthetic drugs into their structures, may contribute to significant progress in the therapy of currently popular skin diseases of various causes [17]. Therefore, the development of biomaterials that can gradually release, from their structures, various natural phytochemicals with antioxidant, anti-inflammatory, moisturizing and anti-aging properties may contribute to a significant improvement in the results of treatment of skin diseases accompanied by inflammation, loss of skin firmness, degradation of elastin and collagen, discoloration and a decrease in the level of skin hydration.

The main aim of this study is to evaluate the potential use of three types of extracts (water (WE), water–ethanol (EE) and water–glycerin (GE)) and hydrogels containing extracts from *C. mas* L. in cosmetic care preparations used in cosmetology and dermatology. This work presents, for the first time, the possibility of incorporating dogwood extracts into the structure of hydrogels based on a natural polymer, which can improve the condition and appearance of the skin. As part of the analyses, a chromatographic analysis of the tested samples was performed, and their antioxidant, cytotoxic and anti-aging properties were assessed. Additionally, their impact on cell migration, transepidermal water loss and skin hydration was assessed.

## 2. Results and Discussion

### 2.1. Chromatographic Analysis of Extracts from Fruit of C. mas L.

**Figure 1:**
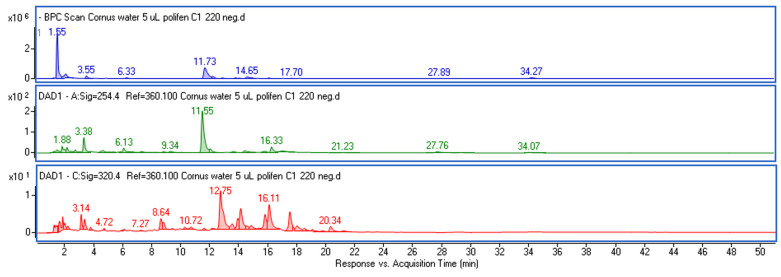
Base peak chromatogram (BPC) obtained in negative ionization mode (blue) and chromatograms registered at wavelengths of 254 nm (green) and 320 nm (red) of water extract from fruits of *Cornus mas*.

The phytochemical composition of the extracts (water (WE), water–ethanol (EE) and water–glycerin (GE)) from the fruits of *C. mas* L. was analyzed using UPLC-DAD-MS. Figure 1 shows examples of MS chromatograms obtained in negative ionization mode and chromatograms registered at wavelengths of 254 and 320 nm. Chromatographic and mass data, along with the results of a quantitative analysis expressed as µg/mL of the extracts, are presented in Table 1. Table S1 (Supplementary Material) includes the MS and UV-VIS spectra of the main identified compounds.

The registered profile was similar to that reported in the literature [18,19]; however, the extracts differed significantly considering qualitative and quantitative composition. It is known that the efficiency of isolating compounds from the plant matrix mainly depends on their chemical nature and polarity, and the physico-chemical properties of the solvent. In general, solvents with low viscosity and surface tension are more effective because they easily penetrate plant material. Our study shows that the water–glycerol (GE) mixture had the lowest extraction efficacy, possibly due to its higher viscosity compared with the other solvents tested. The content of the majority of the identified compounds in GE was significantly lower compared with other extraction mixtures.

In turn, the water extract (WE) contained the highest concentration of iridoids, with loganic acid being the predominant constituent among them. This is due to their better solubility in water than in ethanol. The amount of loganic acid in WE was approximately 1.3- and 2.5-fold higher than in water–ethanol (EE) and GE, respectively. The differences among the other iridoids were only slight.

Considering phenolic acids, the water extract (WE) contained the highest content of gallic, caftaric and protocatechuic acids, with their concentrations being 5.6, 2.9 and 2.6 times higher than in the water–ethanol extract (EE), respectively, and 3.7, 6.0 and 2.8 higher than in GE, respectively. Chlorogenic acid was also present at a higher concentration in WE (8.4-fold increase) and was not found in GE.

In contrast, ellagic acid and quercetin 3-glucuronide were more abundant in EE (approximately 1.6 and 1.5 higher than in WE and approximately 3.0 and 4.8 higher than in GE). EE also contained a higher concentration of anthocyanins, including cyanidin 3-*O*-galactoside and pelargonidin 3-*O*-glucoside [20], than WE, and both components were absent in GE. 

The quantity of quinic acid, a cyclic polyol, in EE and WE did not differ significantly, while it was approximately 1.5 times lower in GE.

### 2.2. Assessment of Antioxidant Activity

#### 2.2.1. DPPH and ABTS Radical Scavenging 

For the purpose of the study, the DPPH and ABTS methods were used to assess the antioxidant activity of three dogwood berry extracts: WE, EE and GE. For each tested sample, the antioxidant properties were expressed as the value of the IC_50_ parameter, which indicates the concentration of the tested sample causing a decrease in the initial concentration of the DPPH or ABTS radical of 50% (Table 2).

As shown by the literature data and chromatographic analyses conducted as part of this work, dogwood berries are characterized by high content of compounds with proven antioxidant properties, such as quinic acid, gallic acid, loganic acid, quercetin 3-glucuronide, ellagic acid and protocatechuic acid [18,21,22]. The higher content of these phytochemicals in WE and EE extracts results in their stronger antioxidant properties compared with the GE extract, as evidenced by the lower IC_50_ values obtained for WE and EE. Hydrogels containing the tested extracts also showed antioxidant activity, but this effect was observed after a longer time, which was probably due to the gradual release of the extracts from the structure of the tested biomaterials. The antioxidant effect of the tested hydrogels was related to the effect of the extracts incorporated in these structures, because the hydrogel base itself did not affect the level of free radicals. These results indicate that the type of extractant used significantly affects the amount and type of biologically active compounds released from dogwood fruit into the solvent during the extraction process. As the obtained results show, extracts prepared on the basis of water, or water and ethanol make it possible to obtain extracts richer in phytochemicals compared with the use of a mixture of water and glycerol. As shown, the activity of the extracts is directly related to the extraction solvent, as it determines the content of active metabolites [23,24]. Commonly used solvents for extracting polyphenols from plant material are water and aqueous mixtures of ethanol and methanol. These solvents ensure the isolation of a wide range of metabolites [25]. 

The results obtained when evaluating antioxidant properties are correlated with the content of polyphenolic compounds determined using UPLC-DAS-MS. 

#### 2.2.2. Intracellular ROS Levels in Skin Cells

**Figure 2:**
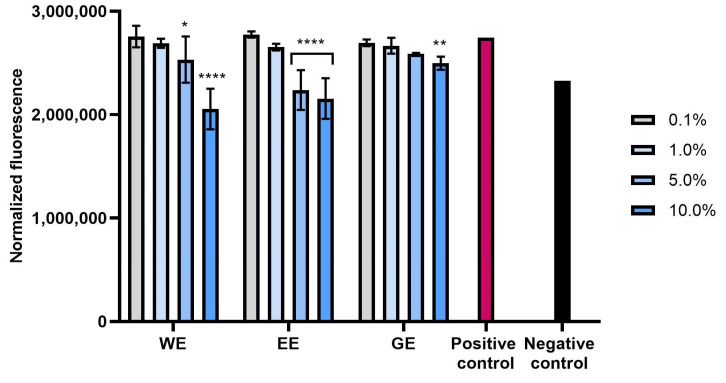
Effect of water extract (WE), aqueous–ethanolic extract (EE) and water–glycerol extract (GE) from *Cornus mas* L. on the intracellular level of reactive oxygen species in fibroblasts (BJ cells). Data are the means ± SDs of three independent experiments in which each sample was tested in 4 replicates. **** *p* < 0.0001, ** *p* = 0.0097, * *p* = 0.0324.

**Figure 3:**
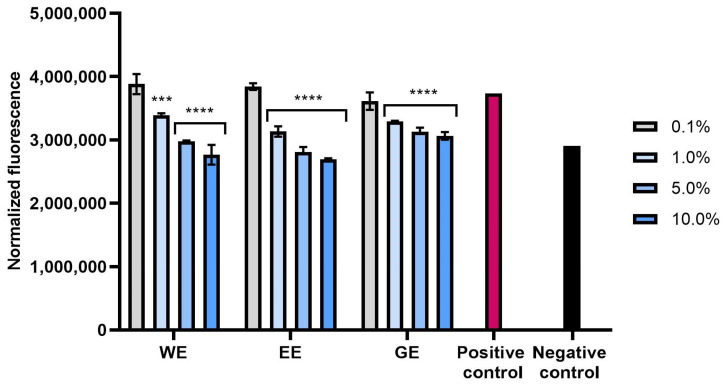
Effect of water extract (WE), aqueous–ethanolic extract (EE) and water–glycerol extract (GE) from *Cornus mas* L. on the intracellular level of reactive oxygen species in keratinocytes (HaCaT cells). Data are the means ± SDs of three independent experiments in which each sample was tested in 4 replicates. **** *p* < 0.0001, *** *p* = 0.0003.

**Figure 4:**
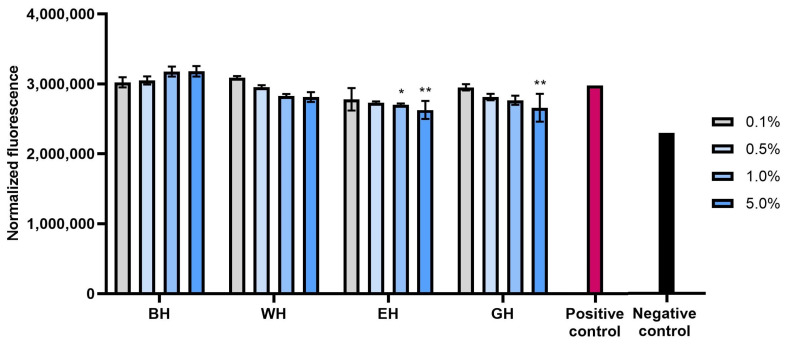
Effect of the base hydrogel (BH) and hydrogels containing water (WH), water–ethanol (EH) and water–glycerol (GH) extracts from *Cornus mas* L. on the intracellular level of reactive oxygen species in fibroblasts (BJ cells). Data are the means ± SDs of three independent experiments in which each sample was tested in 4 replicates. ** *p* = 0.01, * *p* = 0.0272.

**Figure 5:**
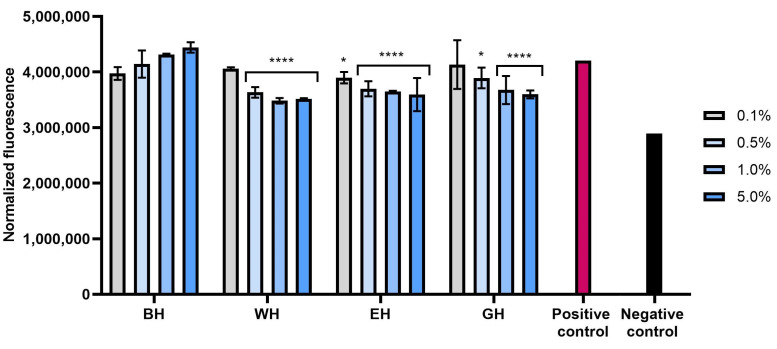
Effect of the base hydrogel (BH) and hydrogels containing water (WH), water–ethanol (EH) and water–glycerol (GH) extracts from *Cornus mas* L. on the intracellular level of reactive oxygen species in keratinocytes (HaCaT cells). Data are the means ± SDs of three independent experiments in which each sample was tested in 4 replicates. **** *p* < 0.0001, * *p* < 0.05.

Excessive amounts of free radicals in skin cells are among the main causes of skin aging and significantly affect its functional disorders, and the formation of wrinkles and skin discolorations [26]. In order to confirm the antioxidant properties of extracts and hydrogels from *C. mas* L., the possibility of reducing the level of reactive oxygen species in both types of skin cells (fibroblasts and keratinocytes) tested after exposure to hydrogen peroxide was also assessed. The analyses performed showed that all types of extracts used have the ability to reduce oxidative stress in both fibroblasts and keratinocytes. This effect was strictly dependent on the concentration used and increased with the increase in the concentration of dogwood extracts (Figure 2, Figure 3, Figure 4 and Figure 5). Although, after using higher concentrations (5%) of the hydrogel base itself, the level of reactive oxygen species (ROS) in the cells increased slightly, the incorporation of dogwood extracts into the structures of the developed hydrogels resulted in a decrease in the level of ROS in these cells. Both in the case of extracts and that of hydrogels, the level of ROS in cells decreased most significantly after the use of WE and EE extracts.

The antioxidant effect of the tested extracts and hydrogels is certainly related to the antioxidant activity of the phytochemicals contained in dogwood fruits. The chemical compounds detected during the chromatographic analysis may support the defense against high levels of ROS by influencing the activity of enzymes such as superoxide dismutase, catalase or glutathione peroxidase [27,28]. The antioxidant effect of the tested extracts may also be the result of inhibiting or quenching free radical reactions in the tested cells. This is largely related to their ability to donate hydrogen atoms, their solubility and chelating properties. In addition to their ability to modulate the activity of the key enzymes mentioned above, phytochemicals contained in plant extracts can also activate or block the transcription of many genes involved in antioxidant defense [29]. The differences in antioxidant activity among the tested samples resulted from different contents of biologically active compounds, which resulted from the use of different extractants during the extraction process.

The use of plant antioxidants in cosmetic or pharmaceutical preparations is extremely important due to the multidirectional action of these compounds [26]. In addition to the documented positive effect on the activity and morphology of skin cells, these compounds significantly influence the stability of skin care preparations. Preventing the oxidation of the compounds contained in these formulations prevents structural and functional changes in cosmetics and extends the shelf life [30]. The presence of compounds with antioxidant activity in the tested extracts from *C. mas* L., confirmed by the chromatographic analysis, suggests that they can be successfully used in multifunctional preparations intended not only to eliminate skin lesions but also to inhibit skin aging processes caused by excessive amounts of free radicals. These compounds can minimize DNA and lipid damage, reduce inflammatory reactions and inhibit excessive activation of the NF-κB or MAPK pathway [26,30]. Additionally, antioxidants can reduce the activation of proteinases responsible for the reconstruction of the extracellular matrix caused by excess reactive oxygen species and inhibit the mechanisms responsible for compromising protein functions [26,31]. 

### 2.3. Cytotoxicity Assessment

The next part of the study evaluated the effects of the tested extracts and model hydrogels on cell viability, metabolic activity and the integrity of skin cell membranes. For this purpose, two tests commonly used for assessing the cytotoxicity of plant materials were used. The Alamar Blue (AB) test was used to assess the impact of the tested extracts and hydrogels on cellular metabolism. This test allows for the quantitative measurement of the cell viability and cytotoxicity of the tested samples by detecting oxidation levels during respiration [32]. On the other hand, the Neutral Red (NR) test assessed the effect of dogwood extracts and hydrogels on cell membrane integrity and the ability to incorporate Neutral Red dye into lysosomes [33]. Cytotoxicity tests were performed on two skin cell lines: keratinocytes (HaCaT) and fibroblasts (BJ).

**Figure 6:**
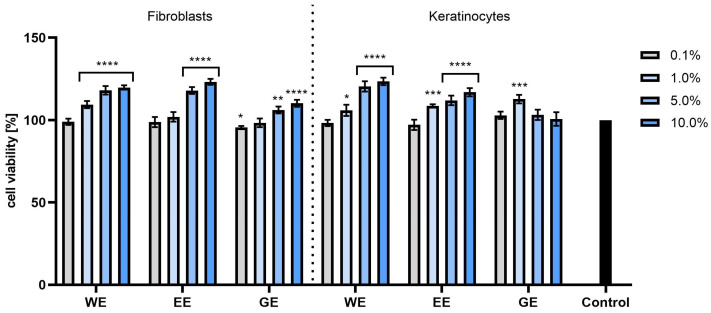
Effect of *Cornus mas* L. extracts (water (WE), water–ethanol (EE) and water–glycerol (GE)) on the reduction of resazurin in cultured fibroblasts and keratinocytes after 24 h of exposure. Data are the means ± SDs of three independent experiments, each consisting of three replicates per test group. **** *p* < 0.0001, *** *p* < 0.001, ** *p* = 0.0025, * *p* = 0.0330.

**Figure 7:**
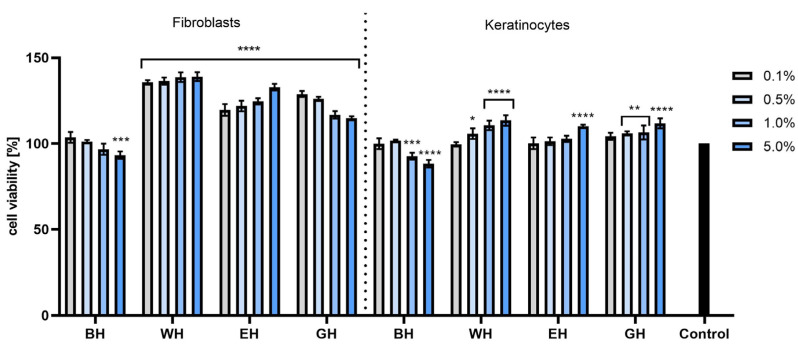
The effect of base hydrogel (BH) and hydrogels (with water extract (WH), aqueous–ethanolic extract (EH) and water–glycerol extract (GH)) with extracts from *Cornus mas* L. on the reduction of resazurin in cultured fibroblasts and keratinocytes after 24 h of exposure. Data are the means ± SDs of three independent experiments, each consisting of three replicates per test group. **** *p* < 0.0001, *** *p* = 0.0009, ** *p* < 0.01, * *p* = 0.0113.

The cytotoxicity analyses performed using the AB test showed that in the case of both HaCaT and BJ cells, all types of extracts had a positive effect on cell proliferation, and the increase in viability depended on the concentration of the tested extracts. It was observed that cells treated with dogwood extracts at the highest tested concentrations (5 and 10%) statistically significantly increased viability and proliferation compared with control cells. When assessing the percentage of viability of the tested cells treated with model hydrogels with extracts, no cytotoxicity was found. Moreover, in the case of fibroblasts, an increase in viability was observed for all preparations at all tested concentrations, and this increase was concentration-dependent. For both fibroblasts and keratinocytes, the most favorable results were observed for WH at concentrations of 1 and 5%, achieving cell viability values of 139.04% ± 2.6 and 113.47% ± 3.1 compared with the control (100%), respectively (Figure 6 and Figure 7). 

**Figure 8:**
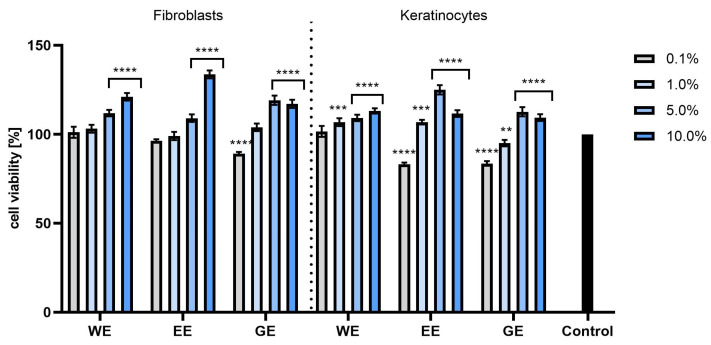
Effect of *Cornus mas* L. extracts (water (WE), water–ethanol (EE) and water–glycerol (GE)) on Neutral Red uptake in cultured fibroblasts and keratinocytes after 24 h of exposure. Data are the means ± SDs of three independent experiments, each consisting of three replicates per test group. **** *p* < 0.0001, *** *p* < 0.001, ** *p* < 0.0069.

**Figure 9:**
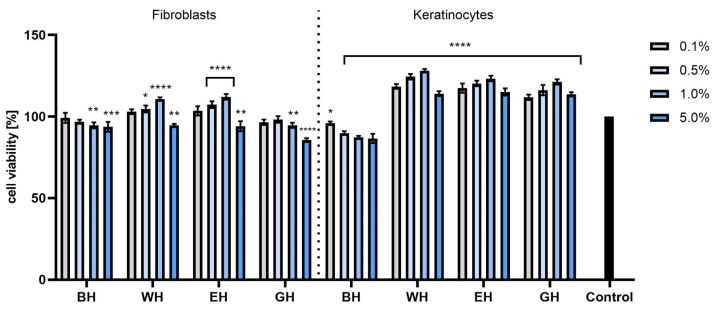
The effect of base hydrogel (BH) and hydrogels (with water extract (WH), aqueous–ethanolic extract (EH) and water–glycerol extract (GH)) with extracts from *Cornus mas* L. on Neutral Red uptake in cultured fibroblasts and keratinocytes after 24 h of exposure. Data are the means ± SDs of three independent experiments, each consisting of three replicates per test group. **** *p* < 0.0001, *** *p* < 0.001, ** *p* < 0.01, * *p* < 0.05.

The cytotoxicity tests performed using the NR test showed that the extracts at concentrations of 1.0 and 5.0% had the most positive effect on the viability of both types of tested cells. The strongest effect was demonstrated by the EE extract, with viability of almost 140% for keratinocytes (for the extract at a concentration of 5%) and almost 130% for fibroblasts (Figure 8). When cells were exposed to hydrogels with extracts from *C. mas* L., it was observed that the incorporation of the extracts into hydrogel structures had a positive effect on the viability of these cells, and this effect was particularly strong in the case of keratinocytes. All tested hydrogels containing extracts statistically significantly increased the viability of these cells, and the viability level was much higher than the values obtained for the base hydrogel (without extracts). In the case of fibroblasts, hydrogels containing water extract (WH) and water–ethanol extract (EH) at a concentration of 1.0% had the most beneficial effect, but when higher concentrations were used, the activity began to decrease (Figure 9).

The extracts tested were rich in active compounds, which may have been responsible for their protective effect on skin cells, which was also confirmed by chromatographic studies (Table 1). Many studies indicate that the iridoid glycosides present in *C. mas* L. extracts have beneficial effects on skin cells. These compounds exhibit strong antioxidant activity; reduce skin damage; and can delay the degradation of the extracellular matrix, which is caused by both ultraviolet radiation and other external factors [9]. Moreover, it has been shown that iridoid glycosides are involved in wound-healing processes by stimulating human dermal fibroblasts involved in anti-inflammatory and immunostimulatory activities [34,35]. Delicato et al. observed an increase in the proliferation of keratinocyte cells (HaCaT) after treatment with acetoside and the dependence of the viability of these cells on the concentration of the compound used and the incubation time [36]. Furthermore, a lack of cytotoxicity towards human fibroblasts was demonstrated when loganic acid was added to the culture medium. Loganic acid also showed a significant collagen synthesis effect by increasing type I and type III collagen in a concentration-dependent manner compared with a negative control, thus improving skin elasticity and showing that it can be used as an active ingredient [37]. Other studies have shown that gold nanoparticles synthesized with polyphenol-rich *C. mas* L. fruit extract exhibited low toxicity and minimal ROS production and did not induce additional DNA damage or an increase in inflammatory cytokine production against human keratinocytes HaCaT and A431, epidermoid cancer cells. In addition, after UVB exposure, the aforementioned formulations exhibited anti-inflammatory effects by modulating the release of certain cytokines [38]. As studies have shown, increasing the viability and metabolism of fibroblast and keratinocyte cells is influenced not only by the extracts themselves but also by model hydrogel formulations with *C. mas* L. extracts implemented into the polymer structure. The positive effects on human skin cells make them potential candidates for future applications in cosmetics and dermatology. 

### 2.4. Scratch Wound Assay

**Figure 10:**
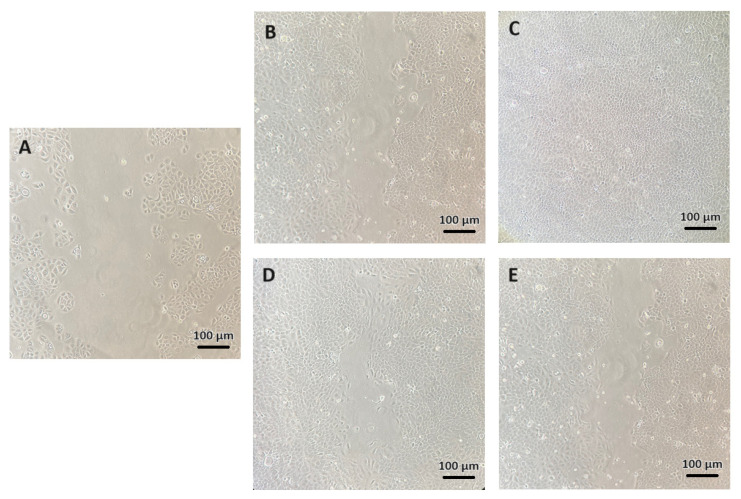
The influence of extracts from *Cornus mas* L. on the migration of keratinocytes. The images show a scratch made on cultured cells using a tip (**A**), control cells (**B**) and cells after treatment (with 1% water extract (**C**), water–ethanol extract (**D**) and water–glycerol extract (**E**)) after 24 h of incubation. Images were taken using an inverted microscope at ×10 magnification (scale bar: 100 μm).

In order to assess the effect of the tested extracts from *C. mas* L. on the migration of keratinocytes, a scratch assay was used. Photos of the cells were taken after 24 h to minimize the role of cell proliferation in closing the scratch created by scratching the cell layer with a tip. These analyses were performed using a lower serum concentration (1% (*v*/*v*) FBS (Fetal Bovine Serum)) in DMEM culture medium to achieve the so-called serum starvation [39]. The assessment of the possibility of cell migration after exposure to the tested dogwood extracts allowed for the assessment of their potential use in tissue regeneration and wound healing [40]. The analyses conducted as part of this study showed that all extracts from *C. mas* L. at a concentration of 1% stimulated the migration of keratinocytes. The greatest migration stimulation was observed with the water extract, and the weakest one, with the water–glycerin extract. In the case of control cells cultured in the DMEM culture medium alone, the migration process was much smaller (Figure 10). In the case of the analyses using the developed hydrogels, increased cell migration was also observed, but the gap was closed after a longer time. This was probably the result of the lower concentrations of extracts that were gradually released from the hydrogel structures. WH had the most pro-migratory effect, followed by EH and GH, and for BH, no significant differences in the migration rate were observed in relation to control cells. The possibility of stimulating migration after the exposure of cells to the biologically active compounds contained in dogwood fruits is consistent with the results previously obtained indicating the pro-migratory potential of extracts and ferments from *C. mas* L. This effect is probably related to the presence, in the tested extracts, of compounds with proven properties that stimulate cell migration, such as quercetin, ellagic acid or chlorogenic acid [41,42,43,44]. The presence of compounds, in dogwood extracts, that can both stimulate proliferation and induce cell migration may contribute to better wound healing and skin regeneration after the use of hydrogels containing these valuable sources of phytochemicals [22]. 

### 2.5. Assessment of Matrix Metallopeptidase Inhibition Using ELISA Method

The aging processes of the skin are closely related to the persistence of its firmness and changes in its structure. These changes are the result of the action of many internal and external factors, including the activity of enzymes that hydrolyze peptide bonds in extremely important proteins in the skin, such as elastin and collagen [45]. The peptide bonds in these proteins are hydrolyzed by two proteolytic enzymes—elastase and collagenase. The possibility of inhibiting or limiting the activity of these enzymes may delay the skin aging process [46]. Therefore, potential inhibitors of the activity of these enzymes are sought by the cosmetics and pharmaceutical industries in order to develop anti-aging cosmetic formulations.

**Figure 11:**
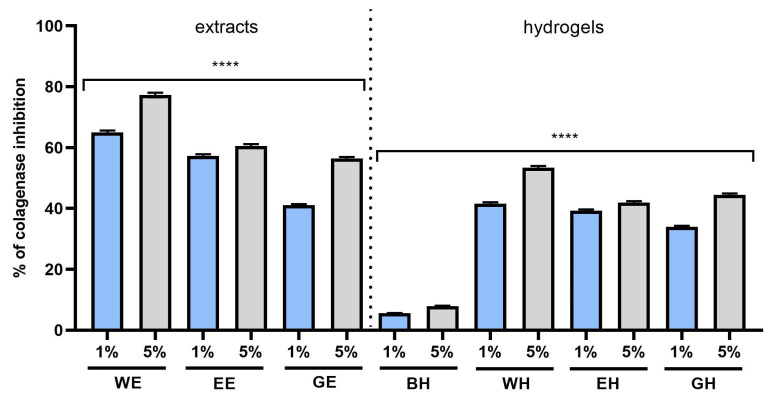
Effect of 1% and 5% extracts (water (WE), water–ethanol (EE) and water–glycerol (GE)) and hydrogels (with water extract (WH), water–ethanol extract (EH) and water–glycerol extract (GH)) on collagenase activity in fibroblasts. Data are the means ± SDs of three independent experiments in which each sample was tested in duplicate. **** *p* < 0.0001.

**Figure 12:**
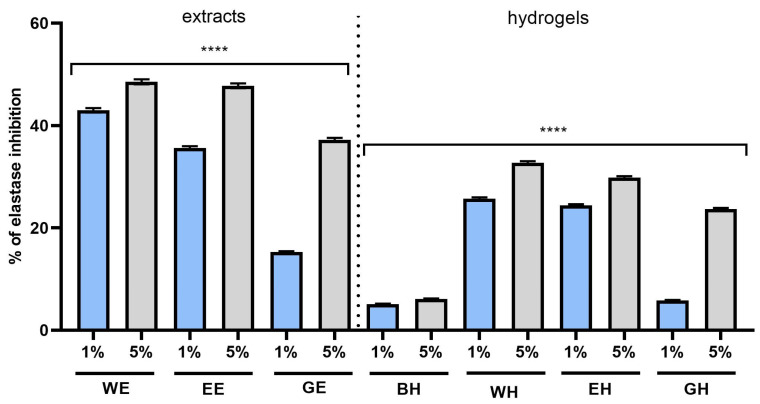
Effect of 1% and 5% extracts (water (WE), water–ethanol (EE) and water–glycerol (GE)) and hydrogels (with water extract (WH), water–ethanol extract (EH) and water–glycerol extract (GH)) on elastase activity in fibroblasts. Data are the means ± SDs of three independent experiments in which each sample was tested in duplicate. **** *p* < 0.0001.

Therefore, one of the goals of this work was to assess and compare the ability of the developed extracts and hydrogels to inhibit the activity of these enzymes. The results of the analyses performed indicated inhibition of the activity of these metalloproteinases by extracts and hydrogels from *C. mas* L., depending on the type of extract and the concentration used. The strongest inhibition of collagenase was observed with 5% WE and EE extracts, for which the inhibition rates were 77.3% and 60%, respectively. In the case of elastase, the inhibition with 5% WE was 48.5%; with EE, 47.7%; and with GE, 37.2%; this indicates stronger anti-elastase properties of the first two extracts. Similar results were observed with the hydrogels, in which the strongest inhibition of both matrix metalloproteinases was noted in the case of the incorporation of the WE extract into the structure of the hydroxyethyl cellulose-based hydrogel (Figure 11 and Figure 12).

Many authors indicate the possibility of inhibiting the activity of collagenase and elastase using the polyphenols present in plant extracts [47,48,49]. Many of these chemicals are present in *C. mas* L. extracts, which resulted in the observed phenomenon of inhibition of the activity of these enzymes by the tested samples. The anti-aging effect of extracts from C.L. is probably the result of the action of the compounds present in these extracts with proven ability to inhibit the activity of collagenase and elastase, such as gallic acid, quercetin 3-glucuronide, quinic acid, ellagic acid and caftaric acid [50,51,52,53].

### 2.6. Transepidermal Water Loss (TEWL) and Skin Hydration Measurements

It is well known that the skin plays an important role in preventing transepidermal water loss. Maintaining homeostasis between the external and internal environment of the skin is important for preventing skin disorders and physical, chemical and bacteriological injuries [40,54]. Moisturizers prevent stratum corneum dehydration when environmental humidity achieves low values. The mechanism of action of these products, which increases water content in the skin, can occur by introducing, into the stratum corneum, hygroscopic substances capable of binding and retaining water. With adequate content of water in the stratum corneum, the skin maintains its barrier function, flexibility and healthy appearance. Hydrophilic polymers have plasticizing and film-forming properties, which make them able to suppress the evaporation of water from the stratum corneum [55]. Increased TEWL is a consequence of impaired skin barrier function. The use of polymeric hydrogels as membrane-forming systems improves skin barrier function and properties, such as skin hydration, due to less pronounced transepidermal water escape from the epidermis [56].

**Figure 13:**
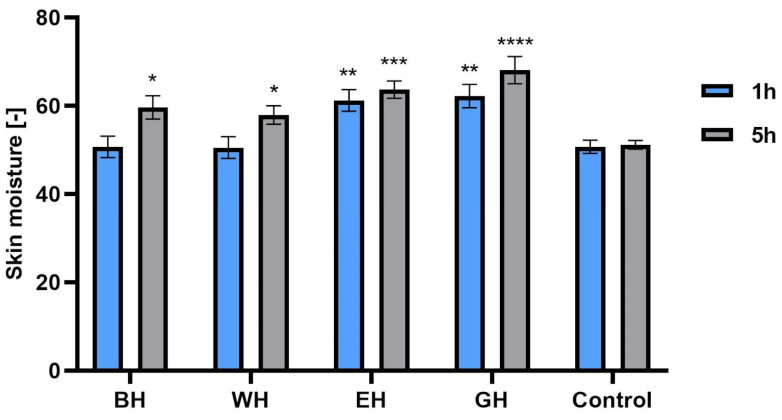
The influence of model hydrogels with *Cornus mas* L. water (WH), water–ethanol (EH) and water–glycerin (GH) extracts on skin hydration. Data are the means ± SDs of three independent measurements. **** *p* < 0.0001, *** *p* < 0.001, ** *p* < 0.01, * *p* < 0.05 compared with the control.

**Figure 14:**
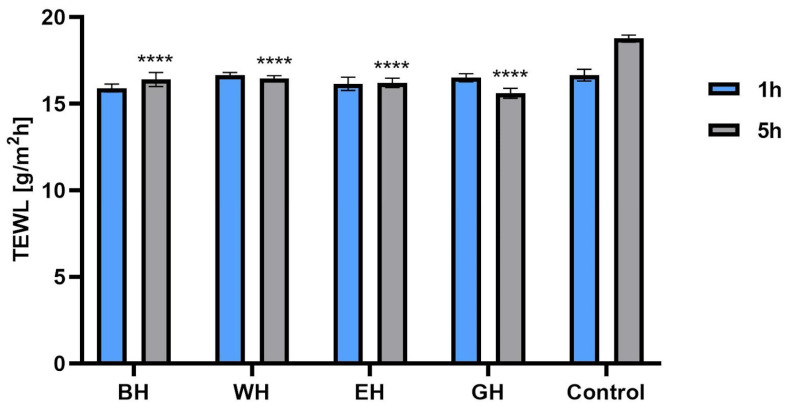
The influence of model hydrogels with *Cornus mas* L. water (WH), water–ethanol (EH) and water–glycerin (GH) extracts on transepidermal water loss (TEWL). Data are the means ± SDs of three independent measurements. **** *p* < 0.0001 compared with the control.

The aim of the study was to evaluate the effect of model hydrogels containing *C. mas* L. extracts on hydration and levels of transepidermal water escape from the epidermis using a corneometer and a TEWAmeter. For this purpose, model hydrogels containing 5% water extract, water–ethanol extract and water–glycerin extract were made. In addition, a hydrogel containing none of the tested extracts was made. The results were statistically compared to the control field (without the tested hydrogel). The measurements were carried out 1 and 5 h after application of the samples to the skin. As shown in Figure 13, for all tested extracts, the formulations showed a more favorable moisturizing effect compared with the base hydrogel. The highest level of hydration compared with the control field was observed 5 h after sample application with hydrogels containing water–glycerin and water–ethanol extracts. When comparing the base and aqueous hydrogels, no statistically significant differences (after 1 h) and small statistical differences (after 5 h) were observed compared with the control field. When examining the level of transepidermal water escape from the epidermis, statistically significant differences were observed with all model hydrogel samples measured 5 h after application of the samples to the skin, where the lowest TEWL value (15.6 g/m^2^h ± 0.29) was observed with the hydrogel containing water–glycerol extract (compared with the control field, the TEWL value was 18.75 g/m^2^h ± 0.21). Measurements taken 1 h after application of the samples showed no statistically significant differences (Figure 14).

*C. mas* L. extracts are rich in active substances, such as polyphenols and iridoids, which may be responsible for moisturizing the skin. A superior moisturizing effect was observed for the hydrogel preparations containing lipid nanoparticles incorporated with aucubin and catalpol. They caused an increase in the epidermis hydration level and a decrease in the TEWL value, contrary to hydrophilic bases without iridoid glycosides [57]. Moreover, hydrogel alone, as a substrate without lipid components in its composition, was not able to maintain TEWL at such a high level of stratum corneum hydration [58]. The most favorable values were obtained with the water–glycerin extract, which may be related to the fact that glycerin is considered a great humectant, as it contains hydroxyl groups in its structure and is able to bind water in the epidermis by forming hydrogen bonds [59]. Furthermore, the water–ethanol extract contained in hydrogel also showed more favorable properties compared with the base hydrogel or with the addition of aqueous extract. Although ethanol can have an adverse effect on skin hydration, in small amounts, it acts as a promoter of penetration of active substances into the epidermis. The authors of this study observed that ethanol increased the solubility of the active compound in the microemulsion, resulting in an increase in the concentration gradient, the main driving force of the transdermal transport process [60,61]. Hydrophilic substances that increase the penetration of active ingredients can act intracellularly and on corneocyte proteins, mainly by interacting with the polar groups of α-keratin. This probably leads to the formation of a system of micropores, through which the transport of compounds via a hydrophilic route can occur. In addition to the interior of corneocytes, in the liquid crystalline structure of the intercellular cement, there are unevenly distributed water areas. Water is maintained through interactions with highly hydrophilic compounds, such as NMF (Natural Moisturizing Factor) components, and polar fragments of peptides and lipids of the cement [62,63].

## 3. Materials and Methods

### 3.1. Materials

2,2-Azino-bis-3-ethylbenzothiazoline-6-sulphonic acid (7 mM ABTS solution; Merck KGaA, Darmstadt, Germany), 2,2-diphenyl-1-picrylhydrazyl (DPPH; Merck KGaA, Darmstadt, Germany), 2′,7′-dichlorodihydrofluorescein diacetate (H_2_DCFDA; Thermo Fisher Scientific, Waltham, MA, USA), acetic acid (CH_3_COOH; ≥99%; Sigma-Aldrich, Poznan, Poland), acetonitrile (MS-grade acetonitrile; Sigma-Aldrich, St. Louis, MO, USA), antibiotics (100 U/mL penicillin and 1000 µg/mL streptomycin; Merck KGaA, Darmstadt, Germany), distilled water (H_2_O; Ultrapure Millipore Direct-Q^®^ 3UV-R; Merck, KGaA, Darmstadt, Germany), DMEM (Dulbecco’s Modification of Eagle’s Medium; Biological Industries, Beit Haemek, Israel), ethanol (C_2_H_5_OH; 96%; Warchem, Zakret, Poland), FBS (Fetal Bovine Serum; Biological Industries, Genos, Lodz, Poland), formic acid (MS-grade acetonitrile; Sigma-Aldrich, St. Louis, MO, USA), HRP conjugate (horseradish peroxidase; Elabscience, Houston, TX, USA), hydrogen peroxide (H_2_O_2_; Merck KGaA, Darmstadt, Germany), hydroxyethyl cellulose (HEC; CHMES, Poznan, Poland), methanol (CH_3_OH; ChemPur, Piekary Śląskie, Poland), Neutral Red solution (NR; 0.33%; Sigma-Aldrich, Poznan, Poland), PBS (phosphate-buffered saline; pH 7.00 ± 0.05; ChemPur, Piekary Ślaskie, Poland), potassium persulfate (2,4 mM; ChemPur, Piekary Śląskie, Poland), resazurin sodium salt (RES; Sigma-Aldrich, Poznan, Poland) Substrate Reagent (3,3′,5,5′-tetrametylobenzydyna; Elabscience, TX, USA), Stop Solution (sulfuric acid solution; Elabscience, TX, USA), RIPA (4-nonylphenol; ethoxylated) buffer (EURx; Gdansk, Poland) and trypsin-EDTA solution (Sigma-Aldrich, Poznan, Poland) were used as received.

### 3.2. Plant Material and Extraction Procedure

Dried *C. mas* L. fruit was obtained from Dary Natury, a Polish company known for producing and distributing herbs in Grodzisk, Poland. Three types of extracts were made: water extract, water–ethanol extract and water–glycerin extract. Before extraction, the plant material was ground with an electric grinder. The study used ultrasonic-assisted extraction according to Yang et al. with minor modifications [64]. The temperature of the extract during extraction changed from 22 °C to 28.7 °C. To prepare the water extract, 20 g of dried dogwood fruit and 100 mL of distilled water were used. To obtain the water–ethanol extract, 20 g of fruit of dogwood, 20 mL of ethanol and 80 mL of distilled water were used, and to prepare the water–glycerin extract, 20 g of dried dogwood fruit, 20 mL of glycerin and 80 mL of distilled water were mixed. The extraction process was carried out with a magnetic stirrer for 1 h; then, the extracts were placed in an ultrasonic bath (Digital Ultrasonic Cleaner) for 30 min at room temperature (approximately 22 °C). The obtained extracts were then filtered using Whatman No. 10 filter paper (Thermo Fisher Scientific, Gothenburg, Sweden). The extracts prepared in this way were used for further research. During the analyses, the extracts were stored at a temperature of approximately +4 °C. The extracts were then stored at −80 °C.

### 3.3. Hydrogel Preparation

The base hydrogel was a 1% aqueous solution of hydroxyethyl cellulose (HEC). For this purpose, 97 mL (92 mL for the hydrogels with extracts) of purified water was measured, and HEC was added and dissolved using a magnetic stirrer (Chemland O20; Hamburg, Germany) at the mixing speed of 250 rpm. After hydrating the polymer, glycerin was added so that its final concentration in the preparation was 2%. Hydrogels with the addition of extracts from *C. mas* L. were prepared in a similar way. Previously prepared extracts were added to the hydrogels at a final concentration of 5%. Four hydrogels were obtained: BH, without the addition of extract; WH, with the addition of aqueous extract of dogwood; EH, with the addition of aqueous–ethanolic extract of *C. mas* L.; and GH, with the addition of water–glycerin dogwood extract.

### 3.4. Determination of Biologically Active Compounds

All standard compounds and reagents, including MS-grade formic acid and MS-grade acetonitrile, were sourced from Sigma-Aldrich (St. Louis, MO, USA). Deionized water was procured using the Ultrapure Millipore Direct-Q^®^ 3UV-R system (Merck, KGaA, Germany).

Chromatographic separation was accomplished using an ultra-high-performance liquid chromatographic system (UHPLC), Infinity Series II, equipped with a DAD detector and an Agilent 6224 ESI/TOF mass detector (Agilent Technologies, Santa Clara, CA, USA). A Titan RP18 column (Supelco, Sigma-Aldrich, Burlington, MA, USA) with dimensions of 10 cm × 2.1 mm i.d. and a particle size of 1.9 µm was employed for the separation. The mobile phase consisted of two components, water containing 0.05% formic acid (solvent A) and acetonitrile containing 0.05% formic acid (solvent B), and it was delivered at a flow rate of 0.2 mL/min. The gradient program applied was as follows: 0–8 min, transitioning from 98% A to 93% A; 8–20 min, maintaining 93% A; 20–40 min, transitioning from 93% A to 80% A; 40–60 min, changing from 80% A to 70% A. The thermostat was set at 30 °C. UV-VIS spectra were recorded in the range of 200 to 600 nm. For mass spectrometry with electrospray ionization (MS-ESI), the parameters included drying gas temperature of 325 °C, drying gas flow rate of 8 L/min, nebulizer pressure of 30 psi, capillary voltage of 3500 V, skimmer voltage of 65 V, and fragmentor voltage of 220 V. Negative ionization mode was utilized for acquiring ions within the mass range of 100 to 1000 *m*/*z*. Identification was carried out by comparing the results with standards or relevant literature data when standards were unavailable.

### 3.5. Determination of Antioxidant Properties

#### 3.5.1. DPPH (1,1-Diphenyl-2-picrylhydrazyl) Radical Scavenging Assay

To determine the antioxidant properties of *C. mas* L. extracts, we used the DPPH radical assay described by Miller et al. [65]. For this purpose, 100 µL aliquots of the tested samples diluted with purified water at concentrations of 0.1, 1.0, 5.0 and 10.0% (*v*/*v*) were placed on a 96-well plate; then, 100 µL of a 4 mM methanolic DPPH solution (Merck KGaA, Darmstadt, Germany) was added to each well and mixed. Distilled water–DPPH solution was used as a control. Absorbance measurements were taken every 5 min for 20 min using a UV-VIS Filter Max spectrophotometer (Aquamate Helton) at a wavelength of 517 nm. Each test sample was performed in 3 repetitions. Using Equation (1), the percentage of DPPH radical scavenging was calculated; then, using a calibration curve and examining the curve equation, IC_50_ was determined, which allowed us to determine which concentration of the extracts caused a 50% decrease in the initial DPPH radical concentration.

(1) $$\%\mathrm{DPPH}\;\mathrm{scavenging}=\frac{\mathrm{Abs}\;\mathrm{control}-\mathrm{Abs}\;\mathrm{sample}}{\mathrm{Abs}\;\mathrm{control}}\times 100$$

#### 3.5.2. ABTS Scavenging Assay

The ABTS Scavenging Assay described by Miller et al. [65] was used to determine the antioxidant properties of the *C. mas* L. extracts. The first step was to prepare a mixture of 7 mM ABTS solution (Merck KGaA, Darmstadt, Germany) and 2.4 mM potassium persulfate in a 1:1 ratio and leave it at room temperature (about 22 °C for 14 h). After 14 h, the solution was adjusted to the absorbance of 1.0 ± 0.04 at a wavelength of 734 nm by diluting it with methanol. The ABTS solution was mixed with extracts diluted with purified water at concentrations of 0.1, 1.0, 5.0 and 10.0% (*v*/*v*) in equal proportions. The absorbance of the obtained samples was measured at a wavelength of 734 nm using a UV/VIS spectrophotometer (Aquamate Helton). Methanol–ABTS solution was used as a control. Each test sample was performed in three repetitions. ABTS radical scavenging was calculated using Equation (2); then, by using a calibration curve and examining the curve equation, IC_50_ was determined, which allowed us to determine which concentration of the extracts caused a 50% decrease in the initial ABTS radical concentration.

(2) $$\%\;\mathrm{of}\;\mathrm{ABTS}\;\mathrm{scavenging}=1-\frac{\mathrm{Abs}\;\mathrm{sample}}{\mathrm{Abs}\;\mathrm{control}}\times 100$$

#### 3.5.3. Determination of Intracellular Levels of Reactive Oxygen Species (ROS)

To determine the ability of the *C. mas* L. extracts and hydrogels to inhibit the intracellular production of reactive oxygen species (ROS) in skin cells, the method by Michalak et al. [66] was used. In this method, the fluorogenic dye 2′,7′-dichlorodihydrofluorescein diacetate (H_2_DCFDA; Thermo Fisher Scientific, Waltham, MA, USA) was used. The first step was to place fibroblasts (BJ) and keratinocytes (HaCaT) in 96-well plates, at the density of 1 × 10^4^ cells/well. Then, the cells were incubated at 37 °C for 24 h. Afterwards, skin cells were treated with extracts and hydrogels dissolved in DMEM for another 24 h. After this time, the tested samples were replaced with 180 µL of 10 µM H_2_DCFDA solution dissolved in DMEM and 20 µL of 500 µM hydrogen peroxide (H_2_O_2_; Merck KGaA, Darmstadt, Germany). Cells not treated with the tested samples, to which only 180 µL of 10 µM H_2_DCFDA solution and 20 µL of 500 µM H_2_O_2_ were added, were used as positive controls. Skin cells not treated with the tested compounds and hydrogen peroxide, to which only the H_2_DCFDA fluorogenic dye solution was added, were used as negative controls. The measurements were performed immediately and after 30, 60 and 90 min of incubation using a microplate reader (Aquamate Helton) at an excitation wavelength of 485 nm and emission λ = 530 nm. This analysis included three independent experiments, with each sample being tested in quadruple.

### 3.6. Cytotoxicity Analysis

#### 3.6.1. Cell Culture

To determine the cytotoxicity of dogwood extracts and hydrogels at different concentrations (0.1, 1.0, 5.0 and 10.0% (*v*/*v*) for extracts and 0.1, 0.5, 1.0 and 5.0% (*v*/*v*) for hydrogels), we used two types of skin cells: BJ fibroblasts (ATCC^®^CRL-2522™; American Type Culture Collection Manassas, VA, USA) and HaCaT keratinocytes (CLS Cell Lines Service, 300493, GmbH, Eppelheim, Germany). Fibroblasts and keratinocytes were grown in DMEM (Dulbecco’s Modified Essential Medium; Biological Industries, Kibbutz Beit-Haemek, Israel) culture medium with L-glutamine supplemented with 10% (*v*/*v*) FBS (Fetal Bovine Serum, Merck KGaA, Darmstadt, Germany) and 1% (*v*/*v*) antibiotic (100 U/mL penicillin and 1000 µg/mL streptomycin, Merck KGaA, Darmstadt, Germany). The skin cells were grown in culture flasks at 37 °C in an incubator in a humidified atmosphere of 95% air and 5% carbon dioxide (CO_2_). When the cells reached appropriate confluence (approximately 70–80%), the culture medium was aspirated, and the cells were rinsed twice with sterile PBS (phosphate-buffered saline; Biological Industries, Kibbutz Beit-Haemek, Israel). Then, the cells were trypsinized to detach them from the bottom of the culture bottle, and fresh DMEM was added. In the next step, the fibroblasts and keratinocytes were separately transferred to 96-well plates at a density of 1 × 10^4^ cells/well and incubated for 24 h in an incubator. This analysis included three independent experiments, with each sample being tested in triplicate.

#### 3.6.2. Alamar Blue Assay

The first test used to assess the viability of the examined skin cells (fibroblasts and keratinocytes) was the Alamar Blue assay, which was carried out according to the procedure described by Page et al. with modifications [67]. After 24 h of incubation, the skin cells with the test extracts and hydrogels of *C. mas* L. dissolved in DMEM (at concentrations of 0.1, 0.5, 1.0 and 5.0% *v*/*v* for hydrogels and 0.1, 1.0, 5.0 and 10.0% *v*/*v* for extracts) were aspirated, and resazurin solution (Merck KGaA, Darmstadt, Germany) at a concentration of 60 µM was added to each well. The untreated cells cultured in DMEM were used as controls. Subsequently, the plates were incubated for 2 h. After this time, the fluorescence was measured at λ = 570 nm with a microplate reader. This analysis included three independent experiments, with each sample being tested in triplicate.

#### 3.6.3. Neutral Red Uptake Assay

The second test used to assess the viability of skin cells was the Neutral Red uptake assay, which was carried out according to the procedure described by Borrenfreund et al. with modifications [68]. After 24 h of incubation, 40 µg/mL of NR dye was added to each well of a 96-well plate containing skin cells with hydrogels (at concentrations of 0.1, 0.5, 1.0 and 5.0% *v*/*v*) and extracts (at concentrations of 0.1, 1.0%, 5.0 and 10.0% *v*/*v*) dissolved in DMEM and incubated for 2 h. Subsequently, the NR dye (Merck KGaA, Darmstadt, Germany) was removed. The cells were washed with sterile PBS and removed. A volume of 150 µL of decolorizing buffer (C_2_H_5_OH/CH_3_COOH/H_2_O, 50%/1%/49%) was added to each well. The absorbance was measured at λ = 570 nm with a microplate reader. This analysis included three independent experiments, with each sample being tested in triplicate.

### 3.7. Scratch Wound Assay

In order to determine the possibility of stimulating the migration of keratinocytes and fibroblasts using dogwood extract and hydrogels, a scratch test was performed based on the procedure previously described by Nizioł-Łukaszewska et al. [69]. The Scratch Wound Assay was performed on skin cells in 6-well flat-bottom plates. After 24 h of incubation, 5% extracts and hydrogels dissolved in DMEM were added. A scratch was made at the center of the well with the tip of a 10 µL pipette, and the plates were incubated again for 24 h. By comparing microscopic images of cells treated with individual samples, cell migration was assessed, and the degree of scratch closure was visually compared. The photos of the cells in the scratch were taken with an inverted microscope at 10× magnification (Nikon Eclipse TS 100-F; Nikon, Warsaw, Poland).

### 3.8. Evaluation of Inhibition of Collagenase and Elastase Activity

In order to determine the ability of *C. mas* L. extracts and hydrogels to inhibit collagenase and elastase activity, a spectrophotometric analysis was performed using a Human COL2α1 ELISA kit and a Human Ne/ELA2 ELISA kit (Elabscience Biotechnology Inc., Houston, TX, USA) based on the instructions provided with the kits. Initially, skin cells (at a density of 1 × 10^4^ cells/well) were seeded into 6-well plates and incubated for 24 h at 37 °C. After this time, the extracts and hydrogels dissolved in DMEM were added (at concentrations of 1.0 and 5.0%), and the plates were incubated again for 24 h at 37 °C. Then, the cells were lysed with RIPA buffer. The cells prepared in this way were subjected to the sandwich ELISA test. Aliquots of 100 µL of standards and tested samples were added to a 96-well plate coated with a specific antibody for human COL2α1 or NE/ELA2 in duplicate and incubated at 37 °C for 90 min. Then, the liquid was removed from the wells using a pipette, and 100 µL of Biotinylated Detection Ab working solution was added to each well; the plates were incubated for 60 min at 37 °C. Fluid was removed from each well, and the wells were washed three times with wash buffer. After this step, 100 µL of HRP conjugate working solution was added to each well, and the plate was incubated again at 37 °C for 30 min. The plate was rinsed again. A volume of 90 µL of Substrate Reagent was added and incubated for 15 min; then, 50 µL of Stop Solution was added, and spectrophotometric measurements were performed at a wavelength of 450 nm using a microplate reader. Inhibition of collagenase and elastase activity was calculated by comparing the level of these proteins in untreated cells and after treatment with the tested samples. Protein levels (collagenase and elastase) were calculated based on the obtained standard curves.

### 3.9. Transepidermal Water Loss (TEWL) and Skin Hydration Measurements

To measure TEWL and skin hydration, 2 cm × 2 cm square areas were marked on the skin of each volunteer’s forearm. Then, 20 µL of the tested hydrogel sample was applied to these areas. The samples were placed around the test area and left to absorb. An area that was not treated with any test sample was considered a control. Measurements were performed after 1 h and 5 h using the TEWAmeter TM 300 and the Corneometer CM 825 probe connected to an MPA adapter (Courage + Khazaka Electronic, Cologne, Germany). The final results were presented as the arithmetic means of 5 independent measurements (from each volunteer) and 20 TEWL measurements.

### 3.10. Statistical Analysis

Values obtained during the experiments performed in this work are presented as means ± standard deviations (SDs). As part of the statistical analysis, analysis of variance (ANOVA) and Dunnett’s intergroup post hoc test were performed. Statistical significance was determined at **** *p* < 0.0001, *** *p* < 0.001, ** *p* < 0.01 and * *p* < 0.05 compared with controls. Statistical analyses were performed using GraphPad Prism 8.4.3 (GraphPad Software, Inc., San Diego, CA, USA).

## 4. Conclusions

The results presented in this work indicate a huge potential for the use of various types of extracts from *C. mas* L. in cosmetology and dermatology. These extracts can be included in cosmetic formulations due to their antioxidant and anti-aging properties, as well as their positive effect on skin hydration and protection against excessive transepidermal water loss. This work indicates the possibility of incorporating dogwood extracts into hydrogel matrices, thereby obtaining efficient carriers of a wide range of phytochemicals present in this plant. The possibility of incorporating extracts containing many biologically active compounds into hydrogel structures may be helpful in the treatment of many skin diseases associated with an excessive number of free radicals, loss of skin firmness and excessive dryness. In the near future, the hydrogels developed as part of this work will be tested in terms of their physico-chemical properties and the possibility of controlled release of active compounds present in dogwood extracts. The possibility of penetration of phytochemicals contained in the tested hydrogels through individual layers of the skin will also be assessed. Due to the constantly growing interest in hydrogels based on herbal extracts, the developed formulations may gain great interest on the cosmetics market.

## Figures and Tables

**Table 1:** Mass data and results of quantitative analysis (means ± SDs) of identified compounds in the extracts from the fruit of *Cornus mas* L.

Rt (min)	Observed Ion Mass [M-H]-/(Fragments)	Δ ppm	Formula	Identified	WE (µg/mL)	EE (µg/mL)	GE (µg/mL)
1.55	191.05677	3.43	C_7_H_12_O_6_	Quinic acid *	45.10 ± 2.55 ^a^	49.83 ± 2.24 ^a^	32.12 ± 1.54 ^b^
3.55	169.01363 (125)	−3.63	C_7_H_6_O_5_	Gallic acid *	36.41 ± 1.91 ^a^	16.51 ± 0.99 ^b^	9.73 ± 0.41 ^c^
4.12	361.07686 (125, 169)	−2.14	C_14_H_18_O_11_	Galloyl-d-sedoheptulose	+	+	+
6.33	153.01905	−1.83	C_7_H_6_O_4_	Protocatechuic acid *	7.21 ± 0.33 ^a^	3.12 ± 0.16 ^b^	2.54 ± 0.15 ^b^
6.80	243.05031 (125, 169)	−2.93	C_10_H_12_O_7_	Galloylglycerol ^1^	4.60 ± 0.28 ^a^	1.82 ± 0.09 ^b^	1.71 ± 0.08 ^b^
11.73	375.13013	1.22	C_16_H_24_O_10_	Loganic acid *	45.51 ± 2.34 ^a^	34.11 ± 1.96 ^b^	17.90 ± 0.98 ^c^
12.93	311.04099 (179, 135)	0.43	C_13_H_12_O_9_	Caftaric acid *	10.32 ± 0.57 ^a^	3.53 ± 0.25 ^b^	1.73 ± 0.09 ^c^
13.83	549.18248 (375, 213)	−0.03	C_23_H_34_O_15_	Loganic acid derivative ^2^	0.98 ± 0.05 ^a^	1.10 ± 0.06 ^a^	0.99 ± 0.04 ^a^
14.15	353.08843 (191, 179)	1.76	C_16_H_18_O_9_	Chlorogenic acid *	2.61 ± 0.13 ^a^	0.31 ± 0.05 ^b^	n.d.
14.65	491.14011 (375)	−1.05	C_20_H_28_O_14_	Loganic acid derivative ^2^	2.12 ± 0.10 ^a^	2.51 ± 0.09 ^a^	2.33 ± 0.12 ^a^
16.13	491.14082 (375)	0.39	C_20_H_28_O_14_	Loganic acid derivative ^2^	1.20 ± 0.06 ^a^	0.65 ± 0.02 ^b^	0.66 ± 0.03 ^b^
17.70	337.09214 (191, 173)	−2.22	C_16_H_18_O_8_	p-coumaroylquinic acid ^3^	1.93 ± 0.08 ^a^	1.21 ± 0.09 ^b^	0.33 ± 0.01 ^c^
17.79	447.09402 (285)	1.64	C_21_H_20_O_11_	Cyanidin 3-*O*-galactoside *	0.41 ± 0.06 ^a^	1.10 ± 0.08 ^b^	n.d.
18.79	431.09891 (269)	1.25	C_21_H_20_O_10_	Pelargonidin 3-*O*-glucoside	0.38 ± 0.04 ^a^	0.93 ± 0.07 ^b^	n.d.
19.04	403.12484	0.63	C_17_H_24_O_11_	Secoxyloganin	+	+	+
27.89	300.99924	0.83	C_14_H_6_O_8_	Ellagic acid *	2.81 ± 0.17 ^a^	4.61 ± 0.30 ^b^	1.51 ± 0.06 ^c^
28.67	477.06655	−1.91	C_21_H_18_O_13_	Quercetin 3-glucuronide *	1.92 ± 0.10 ^a^	2.92 ± 0.14 ^b^	0.61 ± 0.03 ^c^
34.27	541.15619	−0.16	C_24_H_30_O_14_	Cornuside *	1.12 ± 0.08 ^a^	0.44 ± 0.03 ^b^	1.21 ± 0.06 ^a^

WE—water extract; EE—water/ethanol (80/20%) extract; GE—water/glycerol (80/20%) extract; n.d.—not detected; “+”—detected; * identification was confirmed based on standard. Quantification was based on calibration curve for ^1^ gallic acid, ^2^ loganic acid and ^3^ p-coumaric acid. The presence of different letters on the same lines indicates a statistically significant difference (*p* < 0.05) using one-way ANOVA followed by Dunnett’s multiple comparison post hoc test.

**Table 2:** Values of IC_50_ (half-maximal inhibitory concentration) of DPPH and ABTS radical scavenging for three dogwood berry extracts.

IC_50_ (%(*v*/*v*))	Water Extract (WE)	Water–Ethanol Extract (EE)	Water–Glycerin Extract (GE)
	DPPH assay		
	5.62 ± 0.12 ****	6.03 ± 0.09 ****	7.16 ± 0.09 ****
	ABTS assay		
	5.72 ± 0.08 **** (**)	6.69 ± 0.11 **** (**)	

Here, **** *p* < 0.0001 compared with themselves. ** *p* = 0.0013 for WE and EE.

## Data Availability

The data presented in this study are available on request from the corresponding author.

## Supplementary Materials

The following supporting information can be downloaded at: https://www.mdpi.com/article/10.3390/molecules28217384/s1, Table S1: MS and UV-VIS spectra of the main components identified in the extracts from fruit of *C. mas* L.
