# Supplementary material for: Evaluation of the Biological Activity of Hydrogel with Cornus mas L. Extract and Its Potential Use in Dermatology and Cosmetology
Source: Molecules. 2023 Nov 1;28(21):7384. doi: 10.3390/molecules28217384 (PMC10648276; doi:10.3390/molecules28217384)

molecules

Evaluation of the Biological Activity of Hydrogel with *Cornus mas* L. Extract and Its Potential Use in Dermatology and Cosmetology

Martyna Zagórska-Dziok [1,*], Aleksandra Ziemlewska [1], Agnieszka Mokrzyńska [1], Zofia Nizioł-Łukaszewska [1], Magdalena Wójciak [2] and Ireneusz Sowa [2,*]

[1] Department of Technology of Cosmetic and Pharmaceutical Products, Medical College, University of Information Technology and Management in Rzeszow, Sucharskiego 2, 35-225 Rzeszow, Poland; aziemlewska@wsiz.edu.pl (A.Z.); amokrzynska@wsiz.edu.pl (A.M.); zniziol@wsiz.edu.pl (Z.N.-Ł.)
[2] Department of Analytical Chemistry, Medical University of Lublin, Aleje Raclawickie 1, 20-059 Lublin, Poland; magdalena.wojciak@umlub.pl
* Correspondence: mzagorska@wsiz.edu.pl (M.Z.-D.); i.sowa@umlub.pl (I.S.)

Table S1. MS spectra, UV-Vis spectra and the chemical structures of the main components identified in the extracts from fruit of *Cornus mas* L.

Compound	MS spectrum	UV-Vis spectrum	Structure
quinic acid	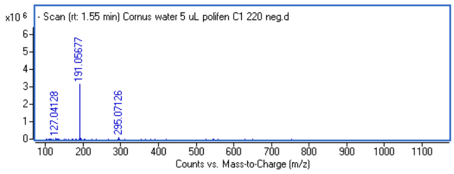		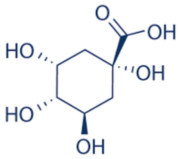
gallic acid	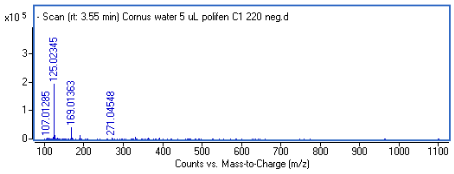	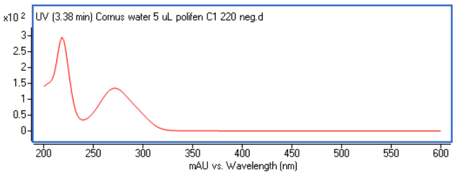	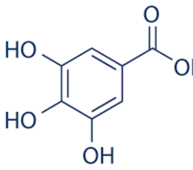
protocatechuic acid	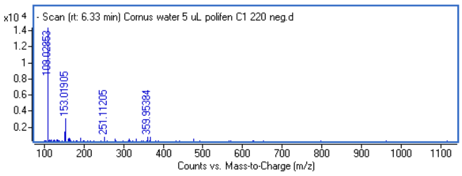	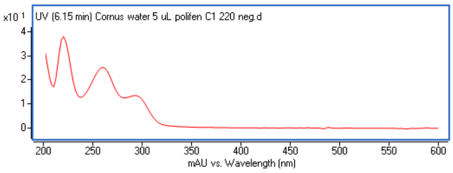	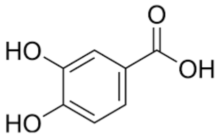
galloylglycerol	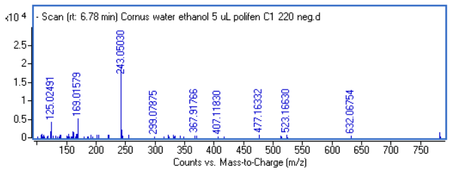	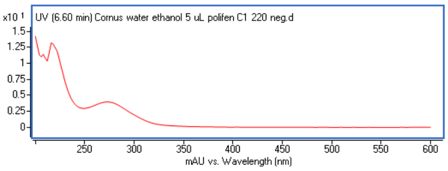	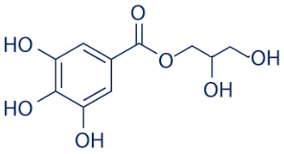
loganic acid	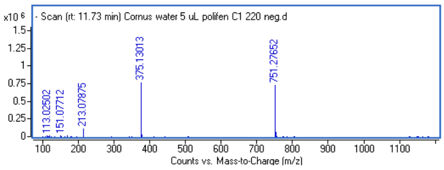	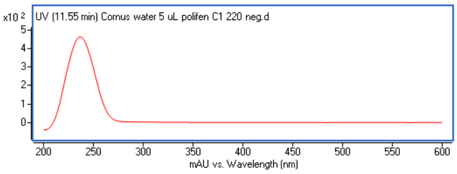	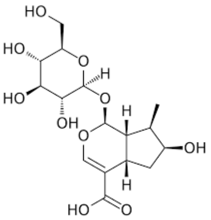

caftaric acid

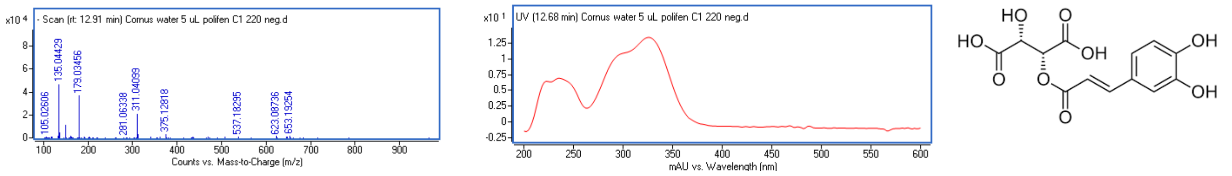

chlorogenic

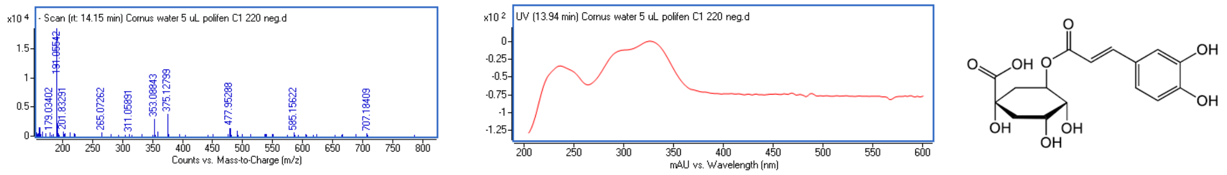

p-coumaroyl-
quinic acid

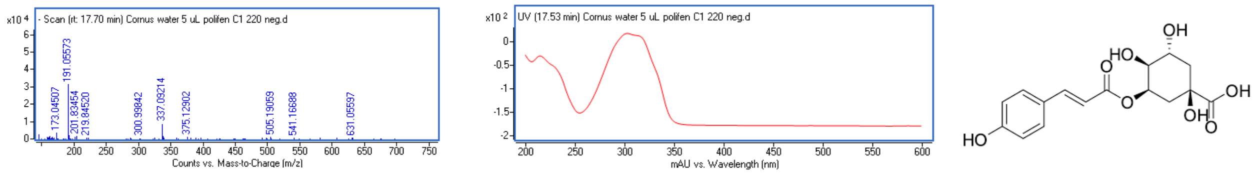

ellagic acid

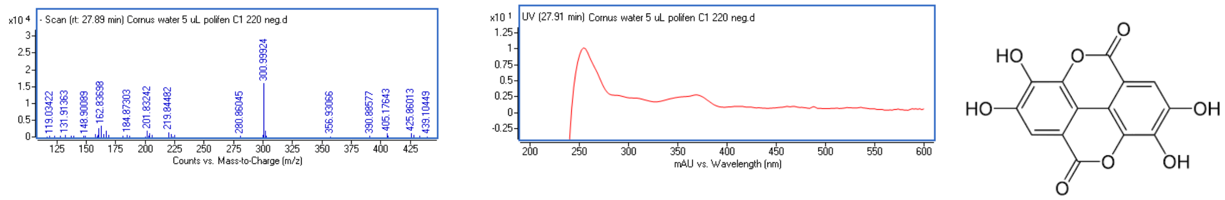

quercetin
3-glucuronide

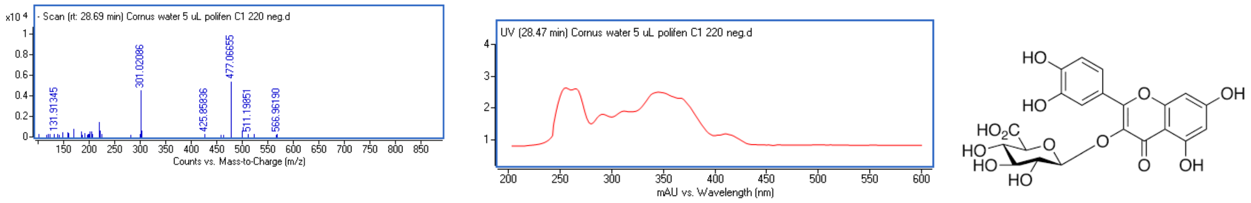

cornuside

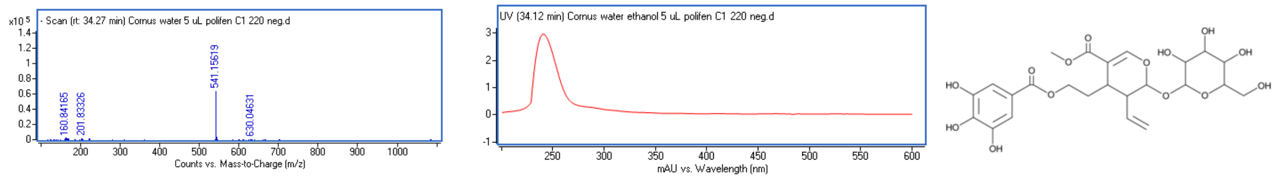

Supplement: Supplementary file 1 [file molecules-28-07384-s001.zip › molecules-2665253-supplementary.pdf]
